# Comprehensive analysis of the prognostic impact and immune implication of KIAA1429 in lung adenocarcinoma

**DOI:** 10.1002/cai2.40

**Published:** 2022-12-16

**Authors:** Lei Guo, Qilin Huai, Bolun Zhou, Jianming Ying, Wei Guo

**Affiliations:** ^1^ Department of Pathology, National Cancer Center/National Clinical Research Center for Cancer/Cancer Hospital Chinese Academy of Medical Sciences and Peking Union Medical College Beijing The People's Republic of China; ^2^ Department of Thoracic Surgery, National Cancer Center/National Clinical Research Center for Cancer/Cancer Hospital Chinese Academy of Medical Sciences and Peking Union Medical College Beijing The People's Republic of China; ^3^ Key Laboratory of Minimally Invasive Therapy Research for Lung Cancer Chinese Academy of Medical Sciences Beijing The People's Republic of China

**Keywords:** KIAA1429, lung adenocarcinoma, m6A methylation, immunotherapy, prognosis

## Abstract

**Background:**

Lung adenocarcinoma (LUAD) is the most common lung cancer worldwide. N6‐methyladenosine (m6A) methylation is a messenger RNA (mRNA) modification that plays a key role in tumor growth, immune microenvironment, and immunotherapy response. This study investigated the expression level, mutation status, prognostic value, and predictive ability for response to anti‐PD‐1 immunotherapy of the m6A methyltransferase KIAA1429 in LUAD.

**Methods:**

This study examined multiple public data cohorts and independent samples from National Cancer Center (NCC) to evaluate the clinical significance and prognostic value of KIAA1429 in LUAD using bioinformatics techniques and immunohistochemical staining. We also evaluated the predictive value of KIAA1429 expression for anti‐PD‐1 immunotherapy efficacy. GSEA analysis was performed using KIAA1429 RNA‐seq data at the tumor tissue level and cellular level to explore the potential molecular mechanism.

**Results:**

In public databases, KIAA1429 was significantly associated with clinicopathological parameters in LUAD patients and had the potential to predict patient prognosis. The mutation characteristics of KIAA1429‐related genes were analyzed and TP53, TTN, CSMD3, and other genes showed high mutation frequencies in LUAD. An independent cohort of 415 samples confirmed that high KIAA1429 expression was significantly associated with poorer prognosis in LUAD patients. Analysis of a small immunotherapy cohort showed that patients with high expression of KIAA1429 had better response after immunotherapy, and the proportion of patients with immunotherapy response was higher in this group.

**Conclusions:**

Our study confirmed that KIAA1429 was highly expressed in LUAD and was significantly associated with poor prognosis. Moreover, KIAA1429 may serve as a potential marker to predict the efficacy of immunotherapy in LUAD.

## Graphical abstract

KIAA1429, an important “writer” of m6A methylation modification, is not only significantly highly expressed in Lung adenocarcinoma (LUAD) but also significantly associated with poor prognosis of LUAD patients. The small sample immunotherapy cohort analysis showed that patients with high expression of KIAA1429 had better response after immunotherapy, and the proportion of immunotherapy response was higher. KIAA1429 is a potential biomarker for predicting prognosis and efficacy of immunotherapy in LUAD patients.

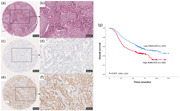

**Abbreviations**
ACC: adrenocortical carcinoma
BLCA: bladder urothelial carcinoma
BP: biological process
BRCA: breast invasive carcinoma
CC: cellular component
CCLE: The Cancer Cell Line Encyclopedia
CNA: copy number alteration
ESCA: esophageal carcinoma
GEPIA: gene expression profiling interactive analysis
GO: Gene Ontology
GSEA: gene set enrichment analysis
IPS: immunophenoscore
KEGG: Kyoto Encyclopedia of Genes and Genomes
LGG: brain lower grade glioma
LIHC: liver hepatocellular carcinoma
LUAD: lung adenocarcinoma
MF: molecular function
NSCLC: non‐small cell lung cancer
PAAD: pancreatic adenocarcinoma
PPI: protein–protein interaction
TCGA: The Cancer Genome Atlas
TCIA: The Cancer Immunome Atlas
THCA: thyroid carcinoma
TIMER: tumor immune estimation resource
TMB: tumor mutation burden
UCEC: uterine corpus endometrial carcinoma

## 1 INTRODUCTION

Approximately 2.2 million new cases of lung cancer were diagnosed in 2020 worldwide, making it the second most common malignancy globally after breast cancer [1, 2, 3]. Lung cancer is divided by pathological type into small cell lung cancer and non‐small cell lung cancer (NSCLC), with the latter representing approximately 85% of all lung cancer cases [4]. Lung adenocarcinoma (LUAD) is the most common pathological type of NSCLC and is characterized by high clinical lethality [5, 6]. Targeted therapy, such as epidermal growth factor receptor–tyrosine kinase inhibitor (EGFR‐TKI), has improved the survival of LUAD patients with EGFR mutation [7]. Immunotherapies, which target the interaction between the tumor and the immune system, have shown initial success in a variety of solid tumors, including lung cancer [8]. PD‐1/PD‐L1 expression, tumor mutational burden (TMB), and interferon signatures are the main predictive biomarkers for the efficacy of immunotherapy [9, 10, 11]. However, these markers have limited ability in predicting immunotherapy efficacy [12]. Therefore, the identification of effective immunotherapy efficacy and prognostic biomarkers will help in the stratification of LUAD patients who will benefit from immunotherapy.

N6‐methyladenosine (m6A) is the most common, conserved, and abundant epigenetic modification of various RNA types, including eukaryotic messenger RNA (mRNA) [13]. Mediated by methyltransferases (“writers”), demethylases (“erasers”), and RNA binding proteins (“readers”), m6A RNA modification is a dynamic and reversible process that has a profound impact on many basic biological processes [14, 15, 16]. Emerging evidence has shown that m6A methylation regulators have cancer‐promoting or inhibiting effects in various malignant tumors [17]. Furthermore, abnormal m6A methylation can significantly affect the immune response during antitumor immunotherapy [18, 19].

KIAA1429 (VIRMA, vir‐Like m6A methyltransferase associated) was first shown to participate in the specific splicing process of the Sxl transcript in fruit flies and later proven to be an important “writer” in m6A modification [20]. KIAA1429 is the largest known component in the complete m6A methyltransferase complex [21]. KIAA1429 is connected to the catalytic core component of METL3/METTL14/WTAP, the scaffold of the methyltransferase complex, and the core component of the RNA substrate to influence the modification and assembly of m6A at specific positions through its N‐KIAA1429 domain [22, 23]. Recent evidence has shown that KIAA1429 is abnormally highly expressed in liver cancer, breast cancer, gastric cancer, and osteosarcoma and that it regulates tumor cell growth and promotes cancer progression through various mechanisms [24, 25, 26, 27]. To the best of our knowledge, there has been no comprehensive study on the prognostic value and immunological efficacy evaluation of KIAA1429 in multiple independent treatment cohorts.

In this study, we conducted a comprehensive analysis of KIAA1429 mutation status and RNA expression and examined the relationship between KIAA1429 expression level and immune responses. We further investigated the prognostic value of KIAA1429 in LUAD.

## 2 MATERIALS AND METHODS

### 2.1 Patients and tissue samples

This retrospective analysis initially enrolled 475 patients (cohort 1) with LUAD from the National Cancer Center/Cancer Hospital, Chinese Academy of Medical Sciences (NCC/CAMS) who underwent R0 surgery between 2006 and 2014. After surgery, the patients were followed up in the outpatient clinic every 3 to 6 months for the first 2 years and then once a year thereafter. Information recorded during follow‐up included medical history, survival status, and observations from physical examination and chest computed tomography (CT). The final follow‐up date was March 4, 2019. The inclusion criteria for patients were as follows: (1) pathologically diagnosed with LUAD and (2) underwent radical surgery R0 resection. The exclusion criteria for the study were as follows: (1) patients who received neo‐adjuvant radiotherapy and/or chemotherapy and (2) patients lacking clinical data. After applying the exclusion criteria, 60 of the 475 patients were excluded from the study. We also included 13 LUAD patients (cohort 2, Supporting Information: Table S1), who received neoadjuvant therapy with immunotherapy combined with chemotherapy.

Patient response to immunotherapy was evaluated using Response Evaluation Criteria in Solid Tumors (RECIST) version 1.1 [28]. All patients underwent comprehensive evaluation with CT or PET‐CT before and after treatment. Before treatment, the lesion measurement requirement for each patient was the sum of the diameters of all target lesions (including the longest diameter of nonlymph node lesions and the short diameter of lymph node lesions) as a reference value for disease baseline. Efficacy after treatment was categorized as follows: CR (complete response, all target and nontarget lesions disappear, all lymph nodes must be nonpathological <10 mm); PR (partial response, at least 30% reduction in the sum of target lesion diameters compared with baseline); and SD (stable disease, target lesion reduction does not achieve PR, enlargement does not achieve PD). Tumor tissues obtained from cohort 2 were used for subsequent reverse‐transcription polymerase chain reaction (RT‐PCR).

This study was conducted in accordance with the Declaration of Helsinki, and it was approved by the Clinical Research Ethics Committee of the NCC/CAMS.

### 2.2 KIAA1429 mRNA expression and mutation profile analyses using The Cancer Genome Atlas (TCGA)

The RNA‐seq expression profiles and corresponding clinical information of 495 patients with LUAD were downloaded from TCGA database using R software (version 3.6.3). The mutation annotation file of 450 LUAD patients was also downloaded to analyze the most significant mutated genes in the KIAA1429 high and low expression groups, and statistical analysis was performed to find the key mutation genes most closely related to KIAA1429. To further explore the prognostic potential of KIAA1429 in different cancer types, we additionally downloaded the genetic and clinical information of patients of multiple cancers including adrenocortical carcinoma (ACC), bladder urothelial carcinoma (BLCA), breast invasive carcinoma (BRCA), esophageal carcinoma (ESCA), brain lower grade glioma (LGG), liver hepatocellular carcinoma (LIHC), pancreatic adenocarcinoma (PAAD), thyroid carcinoma (THCA), and uterine corpus endometrial carcinoma (UCEC) in TCGA. We also explored the association of KIAA1429 with nine representative driver genes (EGFR, ALK, KRAS, BRAF, MEK, MET, PIK3CA, AKT, and LKB1) in the LUAD‐TCGA cohort.

### 2.3 Analysis of KIAA1429 expression using data from the oncomine and Gene Expression Profiling Interactive Analysis (GEPIA) databases

The GEPIA and Oncomine databases are web‐based cancer‐related gene databases that are used to perform tissue‐based analysis of differential expression, survival, and tumor characteristics [29, 30]. We used both GEPIA and Oncomine in the preliminary study of KIAA1429 expression. GEPIA was applied for survival analysis of TCGA LUAD cohort data. The median TPM value was selected as the cutoff.

### 2.4 Tissue microarray (TMA) preparation and IHC of KIAA1429 in LUAD tissues

Tissue blocks from 415 patients with LUAD from the NCC Biobank were used to prepare TMAs, which were then stained by IHC. Briefly, the TMAs were subjected to deparaffinization and then rehydration, followed by a 15‐min treatment with 2 nM HCl and a 10‐min treatment with 100 mM Tris HCl (pH 8.5). The TMAs were then treated with 3% H_2_O_2_ and goat serum at room temperature for 30 min. Following blocking, rabbit anti‐KIAA1429 polyclonal antibody (1:100, HPA002037; Sigma‐Aldrich) was added to the TMAs and the samples were incubated overnight at 4°C. Finally, polyclonal peroxidase‐conjugated anti‐rabbit immunoglobulin G (IgG; Zhongshanjinqiao) was added to the TMAs for a 20‐min incubation at room temperature, in line with the manufacturer's instructions.

### 2.5 Evaluation criteria for immunostaining

KIAA1429 expression of IHC samples was evaluated on the basis of the intensity of cytoplasmic and nucleic staining. The staining intensity was classified as follows: 0 = no staining, 1 = weak, 2 = moderate, and 3 = strong. The percentage of positive cells were scored as follows: 0 = 0%–25% positive staining, 1 = 25%–50% positive staining, and 2 = >50% positive staining. The scores were summed, and samples with a score of ≤1 were classified as the KIAA1429 low expression group and those with a score of ≥2 were classified as the KIAA1429 high expression group.

### 2.6 KIAA1429 immunological value assessment

Tumor Immune Estimation Resource (TIMER, https://cistrome.shinyapps.io/timer/) is a comprehensive analysis website that integrates tumor immunology, clinical information, and genomics [31]. In this study, we used the TIMER “genes” module to explore the correlation of KIAA1429 expression with LUAD tumor‐infiltrating immune cells and corresponding markers. The effect of KIAA1429 copy number alteration (CNA) on the level of immune cell infiltration in the NSCLC tumor microenvironment was assessed using the “SCNA” module. Moreover, PD‐L1 and tumor mutation burden (TMB) data of LUAD patients were downloaded and analyzed from the TCGA database, and immunophenoscore (IPS) data were obtained from The Cancer Immunome Atlas (TCIA) (https://tcia.at/home). IPS score is a novel immune predictor. The sample *Z*‐score of gene expression for all factors (cell types) included in any of the 10 best predictors within each cancer type is color‐coded and divided into four categories. The IPS is calculated on a scale of 0–10 on the basis of the expression of representative genes or genomes from the immune profile [32]. Using the median expression of KIAA1429, differences of PD‐L1 expression, TMB and IPS between different groups were studied.

### 2.7 Quantitative RT‐PCR (qRT‐PCR)

Cancer tissues with at least 70% tumor cells were collected from 13 LUAD patients from an independent immunotherapy cohort in our hospital, and total RNA was extracted from LUAD samples using RecoverAll^TM^ FFPE Total Nucleic Acid Isolation Kit (AM1975; ThermoFisher) following the manufacturer's instructions. Complementary DNA (cDNA) was synthesized using 1 µg total RNA for RT‐PCR. qRT‐PCR was performed on LUAD samples from all immunized cohorts, and the expression of KIAA1429 was calculated and quantified using the $2^{-∆∆C_{t}}$ method. To visualize KIAA1429 expression in each immunotherapy sample, quantified KIAA1429 expression data were log2 transformed. The KIAA1429 primers were KIAA1429 forward TACTTTGAGCCCATTTCTCCTGA and reverse GGAATACTGTCTACTGTTCGTCG. Human β‐actin was used as a control gene for normalization; β‐actin primer sequences were forward CGCGAGAGAAGATGACCCAGATC and reverse GCCAGAGGCGTACAGGGATA. The immunotherapy response status of each sample and original data based on $2^{-∆∆C_{t}}$ KIAA1429 expression levels are shown in Supporting Information: Table S2.

### 2.8 Functional annotation of the KIAA1429 protein–protein interaction (PPI) network and GSEA

The Search Tools for the Retrieval of Interacting Genes/Proteins (STRING) database (http://string-db.org/, version 11.0) is a database for analyzing the interaction of biological genes and proteins [33]. An interaction score of >0.4 was considered to be meaningful, and the top 10 genes most closely associated with KIAA1429 were identified. Kyoto Encyclopedia of Genes and Genomes (KEGG) pathway and Gene Ontology (GO) analyses were used to investigate the PPI network centered on KIAA1429. The enriched molecular function (MF), biological process (BP), and cellular component (CC) terms for genes in the network were visualized.

The Cancer Cell Line Encyclopedia (CCLE, www.broadinstitute.org/ccle) is a database of biological information describing 1457 cell lines and 84,434 genes [34]. To explore the possible molecular mechanism of KIAA1429, RNA‐seq data of 188 lung cancer cell lines in the CCLE were downloaded and compiled.

GSEA was performed on data from TCGA tissue level and CCLE cell level to identify molecular pathways significantly associated with high KIAA1429 expression. Pathways with a *p* < 0.01 and a false discovery rate (FDR) of <0.25 were considered significant.

### 2.9 Statistical analysis

Statistical analysis was performed using SPSS 23.0 (IBM, New York, USA) and R software (version 3.6.3, The R Foundation for Statistical Computing, New Zealand). Statistical significance between KIAA1429 expression and clinicopathological parameters was calculated using non‐parametric tests. For independent samples, the chi‐square test was used to compare differences in KIAA1429 expression and clinicopathological parameters between groups. Kaplan–Meier survival analysis was used to assess the prognostic value of KIAA1429 in LUAD patients, and multivariate Cox regression was used to determine independent prognostic factors. *p* < 0.05 indicated statistical significance.

## 3 RESULTS

### 3.1 KIAA1429 is abnormally expressed in various cancers

**Figure 1:**
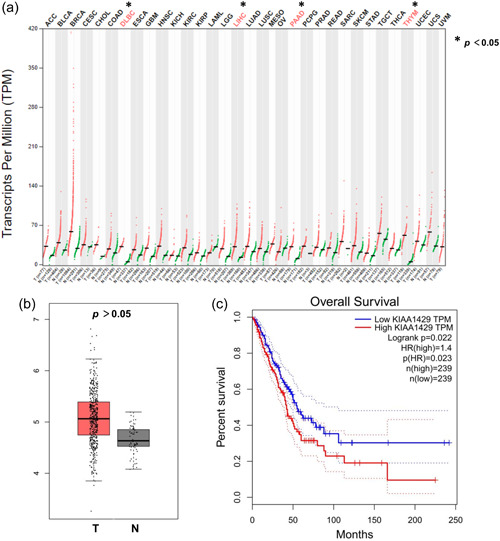
Aberrant expression and prognostic ability of KIAA1429 in a variety of cancers. (a) Expression of KIAA1429 in various cancer tissues and normal tissues in GEPIA. (b) The expression of KIAA1429 in LUAD (red) is higher than that in normal lung tissue (gray) in GEPIA; T, Tumor; N, Normal. (c) High expression of KIAA1429 from GEPIA was significantly (*p* < 0.001) related to the poor overall survival of LUAD patients. GEPIA, Gene Expression Profiling Interactive Analysis; LUAD, lung adenocarcinoma; KIAA1429 (VIRMA), vir‐Like m6A, methyltransferase associated; TCGA, The Cancer Genome Atlas.

Analysis of GEPIA revealed that KIAA1429 was significantly overexpressed in various cancers, including DLBC, LIHC, PAAD, and THYM (Figure 1a). While cancer types such as BLCA, BRCA, and LUAD did not reach statistical differences, a trend of increased KIAA1429 in tumor tissues was observed. KIAA1429 expression was higher in LUAD tissues than in normal tissues (Figure 1b). Kaplan–Meier curve showed that LUAD patients in the high KIAA1429 expression group had significantly lower overall survival than those in the low expression group (Figure 1c). These findings suggest that KIAA1429 expression is abnormally elevated in a variety of cancers.

### 3.2 Identification of independent prognostic factors in TCGA LUAD cohort

Univariate and multivariate Cox logistic regression analyses were performed to further determine the associations between these variables and overall survival in TCGA data set (Table 1). In the univariate model, pT stage (hazard ratio [HR] = 2.455; *p* < 0.0001), pN stage (HR = 2.761; *p* < 0.0001), TNM stage (HR = 2.552; *p* < 0.0001) and KIAA1429 expression (HR = 1.698; *p* < 0.004) were all significantly correlated with OS. Multivariate Cox regression analysis revealed pN stage (HR = 2.303; *p* < 0.0001) and KIAA1429 expression (HR = 1.698; *p* = 0.004) as independent prognostic factors in TCGA LUAD cohort. These findings provide preliminary evidence of the potential prognostic value of KIAA1429 in LUAD patients.

**Table 1:** Univariate and multivariate Cox logistic regression analysis of OS in TCGA cohorts

	Univariate analysis			Multivariate analysis		
Covariates	HR	95% CI	*p* Value	HR	95% CI	*p* Value
Gender (ref. female)	0.927	0.648–1.326	0.677	‐	‐	‐
pT stage (ref. T1–T2)	2.455	1.530–3.937	**<0.000**	1.524	0.907–2.561	0.111
pN stage (ref. N0)	2.761	1.918–3.971	**<0.000**	2.303	1.517–3.495	**0.000**
pM stage (ref. M0)	0.963	0.878–1.056	0.503	‐	‐	‐
TNM stage (ref. I–II)	2.552	1.769–3.683	**<0.000**	1.144	0.917–2.272	0.112
Race (ref. Black)	1.625	0.888–2.972	0.115	‐	‐	‐
Smoked (ref. Never)	0.801	0.550–1.167	0.248	‐	‐	‐
KIAA1429 expression (ref. low)	1.698	1.181–2.441	**<0.000**	1.698	1.177–2.450	**0.004**

*Note*: The bold values indicate the significance of *p* < 0.05.

Abbreviations: CI, confidence interval; HR, hazard ratio; OS, overall survival; TCGA, The Cancer Genome Atlas.

### 3.3 Feature and correlation of molecular mutations based on KIAA1429 expression in LUAD

**Figure 2:**
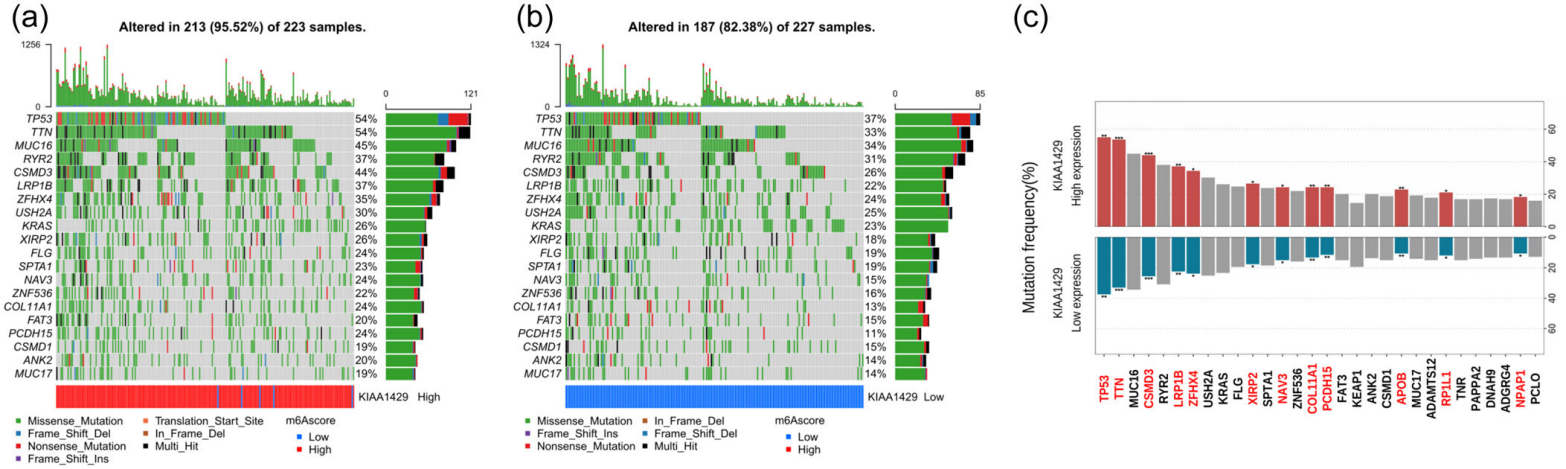
Features of molecular mutation based on KIAA1429 expression in LUAD. Waterfall heatmaps based on annotation information of the top 20 mutant genes in the high (a) and low (b) KIAA1429 mRNA expression groups. (c) Association between gene mutation frequency and KIAA1429 mRNA expression level by chi‐square test. LUAD, lung adenocarcinoma; mRNA, messenger RNA. *p < 0.05, **p < 0.01, ***p < 0.001.

To study the potential mechanism underlying KIAA1429 expression differences in LUAD that may affect the occurrence and development of LUAD, we analyzed the top 20 gene mutation profile significantly related to KIAA1429. We constructed the mutation frequency distribution map of the top 20 mutant genes related to the expression of KIAA1429 (Figure 2a,b). The types of gene mutations mainly included missense mutation, frameshift deletion, and nonsense mutation. Using chi‐square analysis, we searched for key gene mutations, and the mutation frequencies of 12 of them were significantly associated with high and low expression of KIAA1429. The results showed that TP53, TTN, CSMD3, LRP1B, ZFHX4, XIRP2, NAV3, COL11A1, PCDH15, APOB, RP1L1, and NPAP1 gene mutations were significantly enriched in tumor tissues with high and low expression of KIAA1429 (Figure 2c).

We next investigated the correlation of KIAA1429 expression with nine representative driver genes in the LUAD‐TCGA cohort. KIAA1429 expression exhibited weak to moderate correlations with EGFR, ALK, KRAS, BRAF, MEK, MET, PIK3CA, AKT, and LKB1 genes (Supporting Information: Figure S1). The correlation coefficient between KIAA1429 expression and KRAS and PIK3CA genes exceeded 0.5. These results suggest that KIAA1429 may affect the development of LUAD through the above high‐frequency mutated genes, which needs to be further verified by in vivo in vitro experiments.

### 3.4 Relationships between KIAA1429 expression and clinicopathological parameters in the NCC cohort

**Figure 3:**
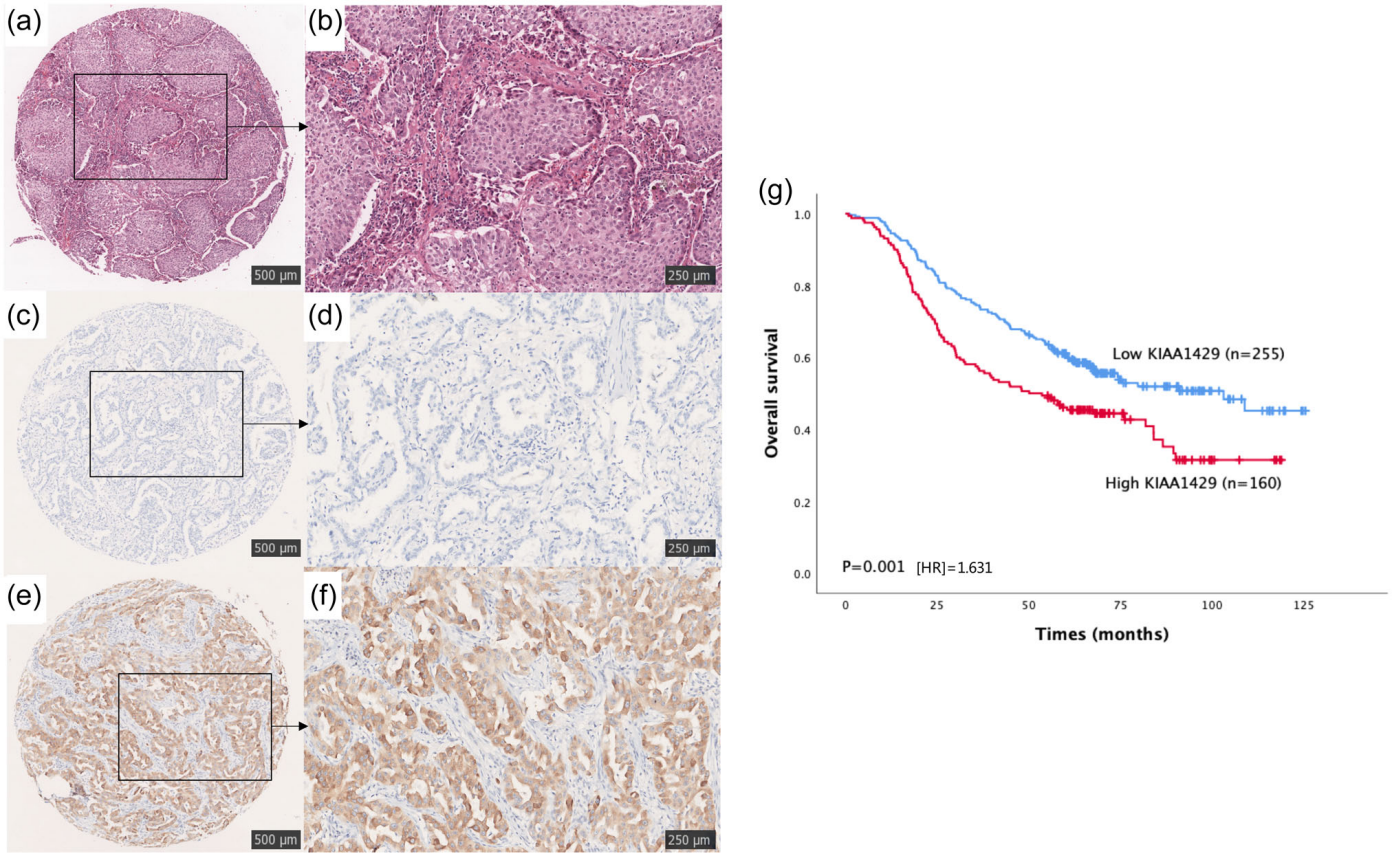
Representative immunohistochemical (IHC) staining of KIAA1429 protein in LUAD TMA. (a, b) H&E staining of LUAD tissue. (c, d) Negative immunohistochemical staining of KIAA1429 in LUAD. (e, f) Positive immunohistochemical staining of KIAA1429 in LUAD. The three pictures on the left were taken at low magnification and the pictures on the right were taken at high magnification. (g) High expression of KIAA1429 is significantly associated with poor overall survival (OS) of LUAD patients in the National Cancer Center of China (NCC) cohort. H&E, hematoxylin and eosin; LUAD, lung adenocarcinoma; TMA, tissue microarray.

We performed IHC staining of 415 samples from the NCC cohort and categorized 160 samples as the high‐expression group. Representative micrographs of hematoxylin and eosin (H&E) and KIAA1429 IHC staining are shown in Figure 3a–f.

As shown in Table 2, KIAA1429 expression was significantly associated with tumor length and T stage (*p* < 0.05) but showed no significant association with age, sex, smoking history, differentiation, N stage, or TNM stage. More samples with high KIAA1429 expression were observed among patients with poorly differentiated and Stage III LUAD. These findings show high expression of KIAA1429 in LUAD and statistically significant differences with some clinicopathological parameters of the patients.

**Table 2:** Correlations between KIAA1429 expression and clinicopathological parameters of 415 patients with LUAD in NCC cohort 1

	Cases (number, %)	KIAA1429 expression		
Category	415 (100%)	Low (255)	High (160)	*p* Value
Age (years)				0.659
≤60	195 (47.0)	122	73	
>60	220 (53.0)	133	87	
Gender				0.210
Male	233 (56.1)	137	96	
Female	182 (43.9)	118	64	
Smoking				0.484
Ever	232 (55.9)	146	86	
Never	183 (44.1)	109	74	
Tumor length (cm)				**0.049**
≤4	237 (57.1)	159	78	
>4	178 (42.9)	96	82	
Differentiation				0.327
Well	57 (13.7)	40	17	
Moderate	159 (38.3)	97	62	
Poor	199 (48.0)	118	81	
T stage				**0.049**
T1	157 (37.8)	104	53	
T2	192 (46.3)	113	79	
T3	41 (9.9)	25	16	
T4	25 (6.0)	13	12	
N stage				0.050
N0	174 (41.9)	111	63	
N1	102 (24.6)	69	33	
N2	139 (33.5)	75	64	
TNM stage				0.087
I	154 (37.1)	97	57	
II	102 (24.6)	70	32	
III	159 (38.3)	88	71	

*Note*: The bold values indicate the significance of *p* < 0.05.

Abbreviations: LUAD, lung adenocarcinoma; NCC, National Cancer Center of China.

### 3.5 Validation of the prognostic potential of KIAA1429 in the NCC cohort

To verify the prognostic potential of KIAA1429 in patients with LUAD, which was demonstrated in the GEPIA database cohort, survival analysis was performed using data of the NCC cohort. The Kaplan–Meier survival curve showed that high KIAA1429 expression was significantly related to a poor prognosis of LUAD (*p* = 0.001, HR = 1.631), indicating that KIAA1429 has prognostic value in LUAD (Figure 3g).

To understand the associations between the clinical variables and OS of patients with LUAD in more detail, univariate and multivariate Cox logistic regression analyses were performed. In the univariate model, age (HR = 1.513; *p* = 0.003), sex (HR = 0.755; *p* = 0.046), smoking (HR = 0.009; *p* = 0.009), tumor length (HR = 2.557; *p* < 0.001), tumor differentiation (HR = 1.758; *p* < 0.001), T stage (HR = 1.919; *p* < 0.001), lymph node metastasis (HR = 2.755; *p* < 0.001), TNM stage (HR = 3.347; *p* < 0.001) and KIAA1429 expression (HR = 1.577; *p* = 0.001) were all significantly correlated with OS. Multivariate Cox regression analysis revealed age (HR = 1.678; *p* < 0.001), tumor length (HR = 1.763; *p* < 0.001), TNM stage (HR = 2.610; *p* = 0.005), and KIAA1429 expression (HR = 1.631; *p* = 0.001) as independent prognostic factors for LUAD patients (Table 3). These findings indicate that KIAA1429 is extremely powerful in predicting the prognosis of LUAD patients.

**Table 3:** Univariate and multivariable analysis of factors associated with overall survival in NCC cohort

	Univariate analysis			Multivariate analysis		
	*p* Value	HR	95% CI	*p* Value	HR	95% CI
Age						
(≤60, >60 years)	**0.003**	1.513	1.150–1.990	**<0.001**	1.675	1.267–2.214
Gender						
(Female, male)	**0.046**	0.755	0.573–0.995	0.653	0.915	0.620–1.349
Smoking						
(Never, ever)	**0.009**	1.431	1.093–1.873	0.442	1.161	0.793–1.700
Tumor length (cm)						
≤4						
>4	**<0.001**	2.557	1.944–3.364	**<0.001**	1.763	1.308–2.375
Differentiation						
(Well/moderate, poor)	**<0.001**	1.758	1.422–2.173	0.126	1.580	0.879–2.838
T stage						
(T1/T2, T3/T4)	**<0.001**	1.919	1.390–2.648	0.849	1.036	0.716–1.500
Lymph node metastasis						
(Negative, positive)	**<0.001**	2.755	2.025–3.749	0.873	1.048	0.587–1.873
TNM stage						
(I/II, III)	**<0.001**	3.347	2.378–4.710	**0.005**	2.610	1.327–5.134
KIAA1429						
Expression (negative, positive)	**0.001**	1.577	1.202–2.068	**0.001**	1.631	1.232–2.158

*Note*: The bold values indicate the significance of *p* < 0.05.

Abbreviations: CI, confidence interval; HR, hazard ratio; NCC, National Cancer Center of China.

### 3.6 Identification of the prognostic potential of KIAA1429 in different cancer types

To further determine the prognostic value of KIAA1429 in different cancer types, we performed survival analysis on other data from other tumors in TCGA database and plotted Kaplan–Meier survival curves. High expression of KIAA1429 was significantly associated with poorer prognosis in patients with nine cancer types including ACC, BLCA, BRCA, ESCA, LGG, LIHC, PAAD, THCA, and UCEC (*p* < 0.05), reflecting its strong prognostic potential (Supporting Information: Figure S2A–I).

### 3.7 Correlation of KIAA1429 with tumor‐infiltrating immune cells and its guiding significance in anti‐PD‐1 immunotherapy

**Figure 4:**
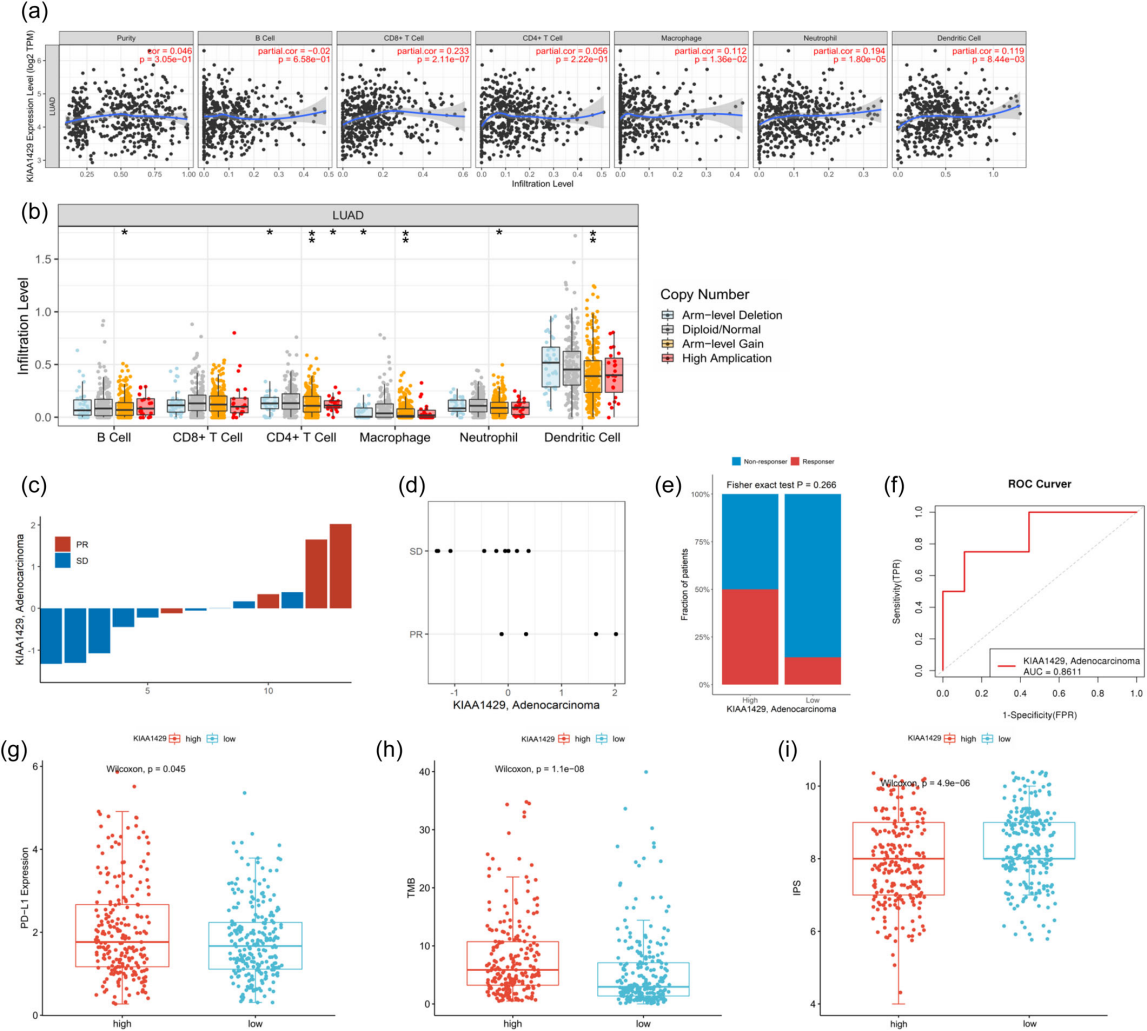
KIAA1429 is associated with tumor‐infiltrating immune cells and predicts anti‐PD‐1 immunotherapy response in LUAD. (a) Correlation of KIAA1429 with major several immune cells and purity in LUAD. (b) Effects of genetic alterations associated with KIAA1429 expression on immune cell infiltration in patients. (c) Histogram of KIAA1429 mRNA expression in LUAD patients treated with anti‐PD‐1 immunotherapy. Blue and red represent stable disease (SD) and partial response (PR), respectively. (d) Scatter plot of KIAA1429 mRNA expression and response to anti‐PD‐1 immunotherapy. (e) Histogram of percentage of immunotherapy response rate and KIAA1429 mRNA expression level. (f) ROC curve of KIAA1429 in predicting response to LUAD immunotherapy. (g) The difference between KIAA1429 and PD‐L1 expression. (h) The difference between KIAA1429 expression and TMB. (i) The difference between KIAA1429 expression and IPS score. LUAD, lung adenocarcinoma; mRNA, messenger RNA; ROC, receiver operating characteristic.

We next explored the correlation between KIAA1429 expression and the level of LUAD tumor immune infiltration using TIMER database. KIAA1429 expression showed a weak positive correlation with LUAD tumor purity, CD8^+^ T cells, CD4^+^ T cells, macrophages, neutrophils, and dendritic cells and a weak negative correlation with B cells (Figure 4a). Correlation analysis of all immune cell‐related gene markers showed that all immune markers were weakly correlated with KIAA1429. The correlation coefficient with NRP1 of DC cells, STAT1 of Th1 cells, STAT3 of Th17 cells, and STAT5B of Treg cells was 0.3 or more (Supporting Information: Table S3). Furthermore, the results on the effect of KIAA1429‐fexpressed somatic CNAs on immune infiltration showed that arm‐level gain, arm‐level deletion, and high amplification in LUAD significantly affected B cells, CD4^+^ T cells, macrophages, neutrophils, and dendritic cell infiltration level, respectively. Between them, the level of dendritic cell infiltration was higher overall, and CD4^+^ T cell somatic mutations were the most frequent (Figure 4b).

We next performed qRT‐PCR on the tumor samples of 13 LUAD patients who underwent immunotherapy in our hospital to investigate the relationship between KIAA1429 mRNA expression and immunotherapy efficacy. Patients with high KIAA1429 expression showed better response to immunotherapy, and two samples with high KIAA1429 expression achieved PR after immunotherapy (Figure 4c,d). Histogram analysis showed that LUAD patients with high expression of KIAA1429 had a higher proportion of response after immunotherapy, but no significant statistical difference was obtained because of the limitation of sample size (*p* = 0.266, Figure 4e). Receiver operating characteristic (ROC) curve analysis showed that KIAA1429 expression had an AUC value of 0.8611 in the immunotherapy cohort, showing good performance in predicting the efficacy of immunotherapy (Figure 4f).

We further analyzed the correlation of widely recognized immunotherapy biomarkers with KIAA1429 expression using TCGA and TCIA databases. PD‐L1 expression, TMB, and IPS were significantly increased in the KIAA1429 high expression group (*p* < 0.05, Figure 4g–i), indicating that KIAA1429 might be a biomarker for identifying patients who may benefit from immunotherapy.

### 3.8 GSEA and functional annotation of the KIAA1429 PPI network

**Figure 5:**
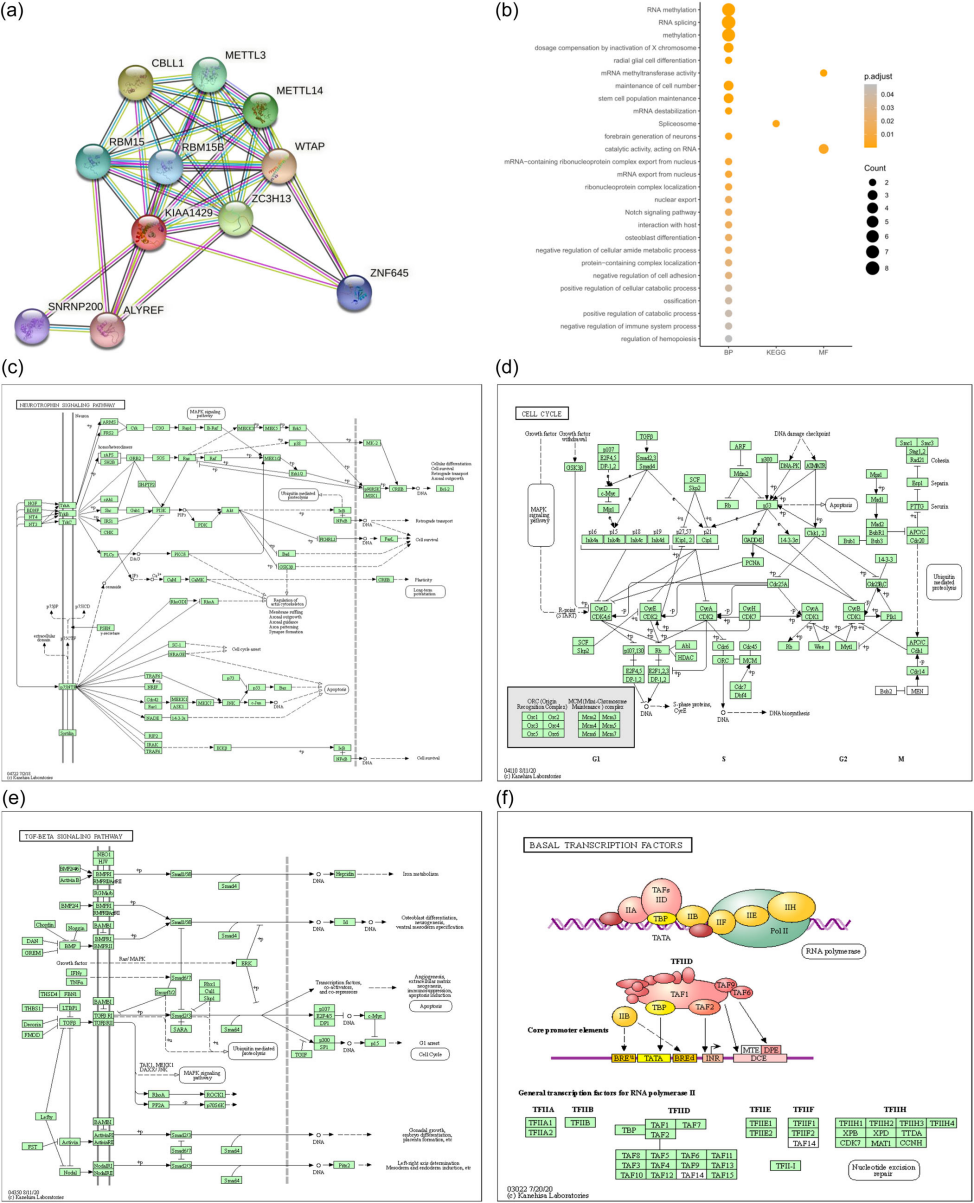
KIAA1429‐related PPI network construction and biological function analysis. (a) PPI network constructed by 10 genes (CBLL1, METTL3, METTL14, RBM15, RBM15B, WTAP, ZC3H13, ZNF645, SNRNP200, and ALYREF genes) significantly related to KIAA1429. (b) GO terms including biological process (BP) and molecular function (MF) and KEGG pathways analysis. GSEA analysis of 188 lung cancer cell lines from CCLE yields the (c) neurotrophin signaling pathway, (d) cell cycle, (e) TGF‐BETA signaling pathway, and (f) basal transcription factors. CCLE, The Cancer Cell Line Encyclopedia; GO, Gene Ontology; GSEA, gene set enrichment analysis; KEGG, Kyoto Encyclopedia of Genes and Genomes; PPI, protein–protein interaction.

To investigate the relationships between 10 molecules that were significantly related to KIAA1429, a PPI network was constructed using the STRING database. The 10 molecules were CBLL1, METTL3, METTL14, RBM15, RBM15B, WTAP, ZC3H13, ZNF645, SNRNP200, and ALYREF (Figure 5a). GO and KEGG functional enrichment analyses revealed that the BPs of KIAA1429 mainly included RNA methylation, maintenance of cell number, and stem cell population maintenance (Figure 5b).

To determine the potential molecular mechanism of KIAA1429 in LUAD, the Hallmark gene set was used to perform GSEA. The results showed that in samples expressing high levels of KIAA1429, many gene sets were positively enriched, and G2M checkpoint, E2F targets, MYC targets, and mitotic spindle were the top four most relevant BPs (Supporting Information: Figure S3A–D). The top 10 enriched pathways are shown in Table 4, and the top 100 genes that were significantly positively or negatively correlated with KIAA1429 expression are presented in Supporting Information: Figure S3E.

**Table 4:** Top 10 biological processes enriched in lung adenocarcinoma based on KIAA1429

Rank	Name of pathway	ES	NES	NOM *p* value	FDR *q* value	FWER *p* value
1	G2M_CHECKPOINT	0.75	3.53	0.000	0.000	0.000
2	E2F_TARGETS	0.74	3.51	0.000	0.000	0.000
3	MYC_TARGETS_V1	0.58	2.77	0.000	0.000	0.000
4	MITOTIC_SPINDLE	0.54	2.56	0.000	0.000	0.000
5	MYC_TARGETS_V2	0.59	2.36	0.000	0.000	0.000
6	SPERMATOGENESIS	0.47	2.16	0.000	0.000	0.000
7	MTORC1_SIGNALING	0.44	2.11	0.000	0.000	0.000
8	UNFOLDED_PROTEIN_RESPONSE	0.42	1.90	0.000	0.000	0.001
9	PROTEIN_SECRETION	0.30	1.32	0.018	0.070	0.234
10	GLYCOLYSIS	0.21	1.04	0.333	0.545	0.897

Abbreviations: ES, enrichment score; FDR, false discovery rate; FWER, familywise‐error rate; NES, normalized enrichment score; NOM, nominal *p* value.

RNA‐seq data from 118 lung cancer cell lines from the CCLE database were also used to perform GSEA. As shown in Figure 5c–f, GSEA analysis revealed KIAA1429 was significantly related to several signaling pathways, including the neurotrophin signaling pathway, cell cycle, TGF signaling pathway, and basal transcription factors. The top 10 signaling pathways enriched in KIAA1429 in the 118 lung cancer cell lines from the CCLE database are shown in Table 5. These findings identified the possible involvement of KIAA1429 in molecular pathways affecting LUAD progression.

**Table 5:** Top 10 KEGG pathways enriched in 118 lung cancer cell lines from CCLE based on KIAA1429

Rank	Name of pathway	ES	NES	NOM *p* value	FDR *q* value	FWER *p* value
1	NEUROTROPHIN_SIGNALING_PATHWAY	0.44	1.72	0.008	0.747	0.375
2	CELL_CYCLE	0.50	1.70	0.021	0.442	0.422
3	TGF_BETA_SIGNALING_PATHWAY	0.41	1.61	0.010	0.639	0.641
4	BASAL_TRANSCRIPTION_FACTORS	0.51	1.54	0.036	0.740	0.762
5	NON_SMALL_CELL_LUNG_CANCER	0.40	1.45	0.037	1.000	0.891
6	GAP_JUNCTION	0.37	1.42	0.045	1.000	0.926
7	HOMOLOGOUS_RECOMBINATION	0.52	1.42	0.136	0.926	0.926
8	CHRONIC_MYELOID_LEUKEMIA	0.37	1.41	0.067	0.835	0.931
9	MISMATCH_REPAIR	0.55	1.40	0.152	0.797	0.939
10	INOSITOL_PHOSPHATE_METABOLISM	0.38	1.40	0.059	0.736	0.939

Abbreviations: ES, enrichment score; FDR, false discovery rate; FWER, familywise‐error rate; KEGG, Kyoto Encyclopedia of Genes and Genomes; NES, normalized enrichment score; NOM, nominal *p* value.

## 4 DISCUSSION

The mechanism of m6A methylation in lung cancer has been uncovered through ongoing research, and many m6A methylation regulators have been shown to play an important role in this disease. For example, high expression of the m6A demethylase FTO in lung squamous cell carcinoma indicates a poor patient prognosis. Furthermore, FTO promotes cancer progression by regulating the expression of MZF1, and knockdown of FTO inhibits cell proliferation and invasion [35]. METTL3 increases the expression of EGFR and TAZ and promotes the growth, survival, and invasion ability of human lung cancer cells; it also plays a role in promoting the translation of lung cancer oncogenes [36]. Recent studies have shown that m6A modulators can not only regulate tumor proliferation and migration but also significantly affect the immune response during antitumor immunotherapy. Evidence has shown that m6A modulators are promising biomarkers for predicting immune efficacy and enable improved immunotherapy outcomes in cancer patients [37, 38]. However, research on the association of m6A methylation with tumor immunity and prognosis remains sparse, especially in terms of investigating specific m6A regulators, like the “writer” KIAA1429.

In this study, using public database bioinformatics analysis, we found that KIAA1429 was significantly associated with clinicopathological parameters in LUAD patients and had the potential to predict patient outcomes. We also performed a detailed analysis of the mutational characteristics of KIAA1429‐related genes in LUAD and found that multiple genes such as TP53, TTN, and CSMD3 genes showed a higher mutation frequency in KIAA1429‐expressing tumor tissues. While the top 20 mutated genes did not change in the two groups of samples with high and low KIAA1429 expression, the percentage of mutations and mutation types were different. Thus, KIAA1429 expression differences may affect the mutation of certain genes and this possibility needs to be verified by more in‐depth studies. We then used an independent cohort to validate the prognostic value of KIAA1429 for LUAD patients. We further conducted survival analysis in multiple cancer cohorts including ACC, BLCA, BRCA, ESCA, LGG, LIHC, PAAD, THCA, and UCEC patients in TCGA and found that high expression of KIAA1429 was significantly associated with poorer OS in patients.

Given the potential relationship between m6A methylation regulators and tumor immunity, we also analyzed the extent to which KIAA1429 is involved in tumor immune cell infiltration and immunotherapy response in LUAD. Although KIAA1429 showed weak correlation with tumor purity, CD8^+^ T cells and other major immune cells, it was still observed that intracellular arm‐level proliferation, arm‐level deletion, and high amplification were able to influence the level of immune cell infiltration in LUAD. Analysis of an independent immunotherapy cohort from our hospital showed that patients with high expression of KIAA1429 showed better efficacy response and a higher frequency of immunotherapy response after receiving immunotherapy. ROC curve analysis showed that KIAA1429 exhibited good predictive performance in predicting the efficacy of immunotherapy. We also explored the relationship between classical immunotherapy response biomarkers and KIAA1429 expression. Significant increases in PD‐L1, TMB, and IPS were observed in patients with high KIAA1429 expression, which was in line with our results. Previous studies showed that the m6A RNA methyltransferases METTL3 and METTL14 enhance the response of colorectal cancer to immunotherapy by regulating the tumor immune microenvironment and negatively correlate with the STAT1 signaling pathway; these factors are potential new targets for antitumor immunotherapy [39]. The m6A “reader” YTHDF1 modulates melanoma immune responses by enhancing antigen‐specific CD8^+^ T cell antitumor responses [18]. Yang et al. further demonstrated that FTO increased m6A methylation levels in PD‐1, CXCR1, and SOX10 mRNA and inhibited melanoma growth. Selective blockade of FTO restores IFN‐γ responses and makes anti‐PD‐1 therapy more sensitive [40]. KIAA1429 is not only potentially related to immunity but is also an upstream gene of the key glycolytic enzyme HK2, which enhances the stability of HK2 in an m6A‐dependent manner, thereby promoting tumor glycolysis [41]. Recent studies have shown that KIAA1429 promotes colorectal cancer progression by inhibiting WEE1 expression in an m6A nondependent manner [42]. To explore the possible molecular functions of KIAA1429 in LUAD, we performed GSEA on data from tissue samples and cell samples from multiple databases and obtained enriched biological pathways. These studies provide a rich theoretical basis for further exploration of the mechanism of action of KIAA1429 in LUAD.

Although this study goes some way towards revealing the potential of KIAA1429 in the evaluation of immunotherapy efficacy and prediction of prognosis, it still has some limitations. First, although our hospital's large‐sample cohort supported the research conclusions, a single‐center study can also entail biases. We attempted to expand the size of the LUAD immunotherapy sample, but the sample size included in the immunotherapy cohort was still small. Additionally, the molecular mechanism of KIAA1429 in LUAD will need to be verified in vitro and in vivo.

In conclusion, our study confirmed that high expression of KIAA1429 is associated with poor prognosis in LUAD patients. Patients with high expression of KIAA1429 had better response to immunotherapy and a higher proportion of immunotherapy response, indicating KIAA1429 showed excellent predictive performance in predicting the efficacy of LUAD immunotherapy. Therefore, KIAA1429 is a potential immunotherapy efficacy evaluation and prognostic biomarker.

## AUTHOR CONTRIBUTIONS

**Lei Guo**: Conceptualization (equal); Investigation (equal); Methodology (equal); Resources (equal); Validation (equal); Writing – original draft (equal). **Qilin Huai**: Data curation (equal); Formal analysis (equal); Investigation (equal); Validation (equal); Visualization (equal); Writing – original draft (equal). **Bolun Zhou**: Data curation (equal); Investigation (equal); Methodology (equal); Resources (equal); Validation (equal); Visualization (equal). **Jianming Ying**: Data curation (equal); Methodology (equal); Resources (equal); Validation (equal); Visualization (equal). **Wei Guo**: Conceptualization (equal); Funding acquisition (equal); Project administration (equal); Resources (equal); Software (equal); Supervision (equal); Validation (equal); Visualization (equal); Writing – original draft (equal).

## CONFLICT OF INTEREST

The authors declare no conflict of interest.

## ETHICS STATEMENT

This study was conducted in accordance with the Declaration of Helsinki, and it was approved by the Clinical Research Ethics Committee of the NCC/CAMS (NCC2020C‐211). This study has not been published in any other journal, and all authors agree to publish this study in *Cancer Innovation*.

## INFORMED CONSENT

All participants provided informed consent before participating in this study.

## Supporting information

Supporting information.

Supporting information.

Supporting information.

Supporting information.

Supporting information.

Supporting information.

## Data Availability

All data generated or analyzed on the basis of public databases in this study were obtained from the TCGA, TIMER, TCIA and CCLE databases, and relevant descriptions and website links are included in this article. Independent cohort experimental data can be obtained from the corresponding author upon reasonable request.
