# Supplementary figures and images for: Comprehensive analysis of the prognostic impact and immune implication of KIAA1429 in lung adenocarcinoma
Source: Cancer Innov. 2022 Dec 16;1(4):328–43. doi: 10.1002/cai2.40 (PMC10686173; doi:10.1002/cai2.40)

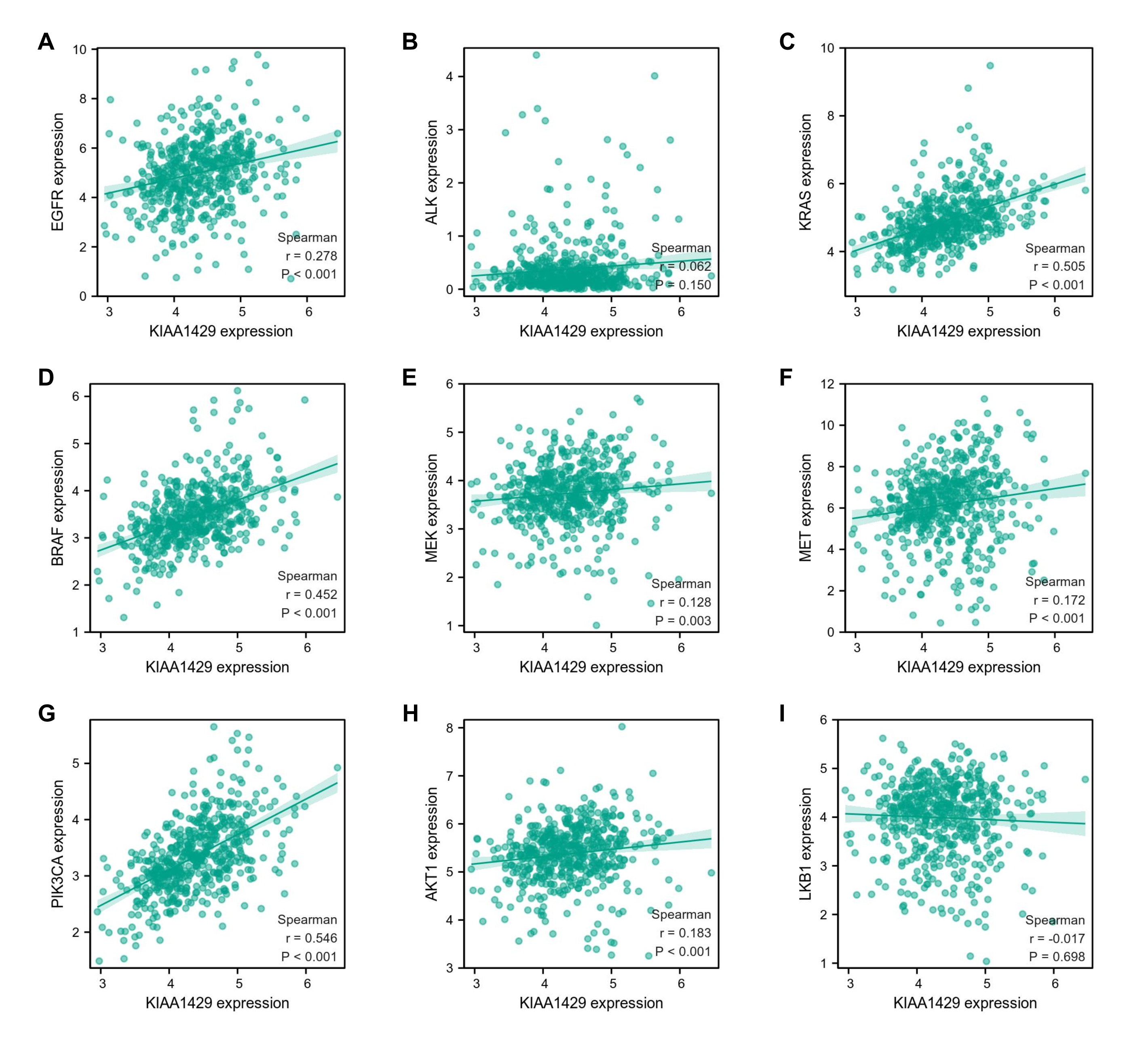

Supplement: Supplementary file 1 — Supporting information. [file CAI2-1-328-s005.tif]

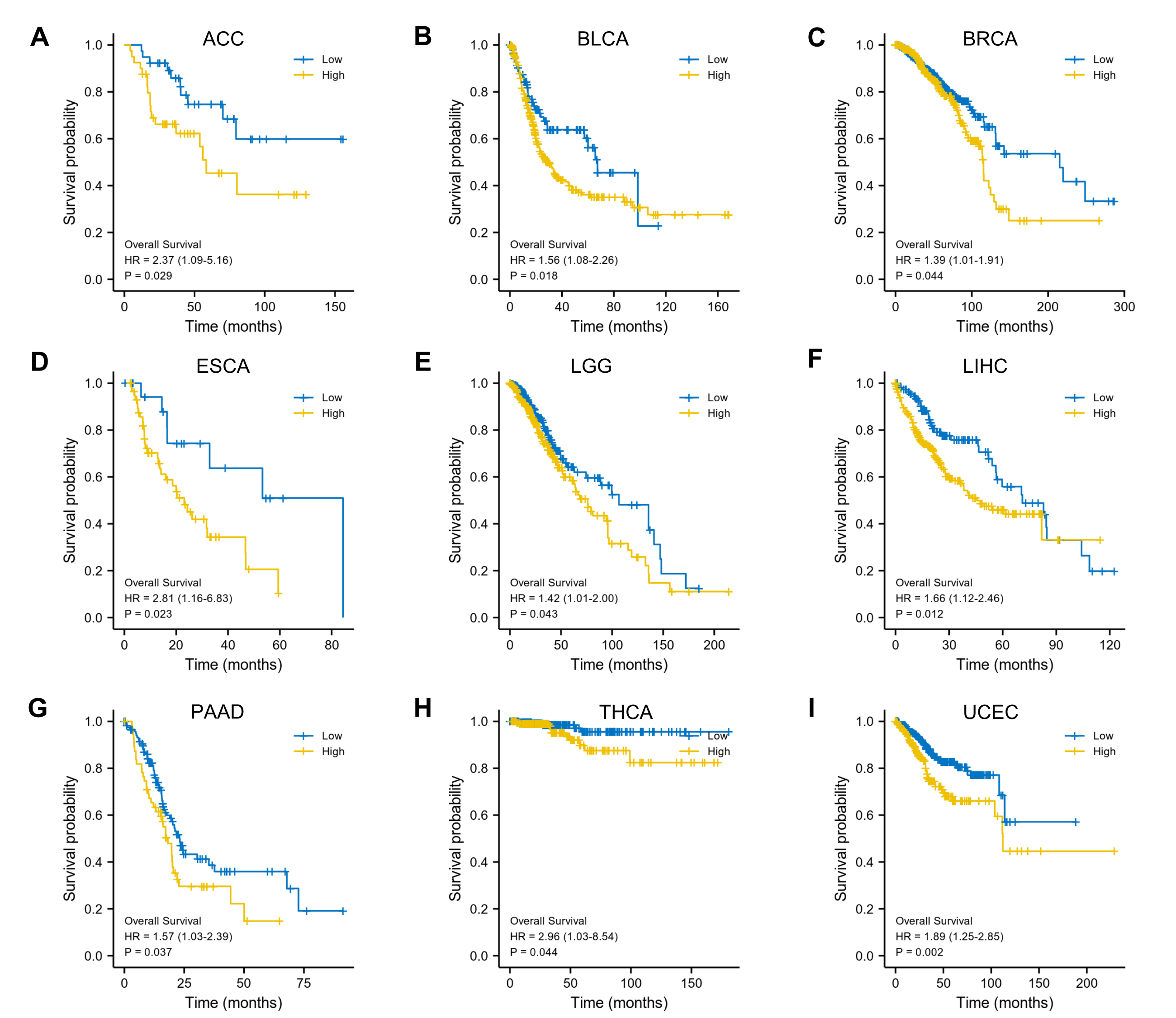

Supplement: Supplementary file 2 — Supporting information. [file CAI2-1-328-s002.tif]

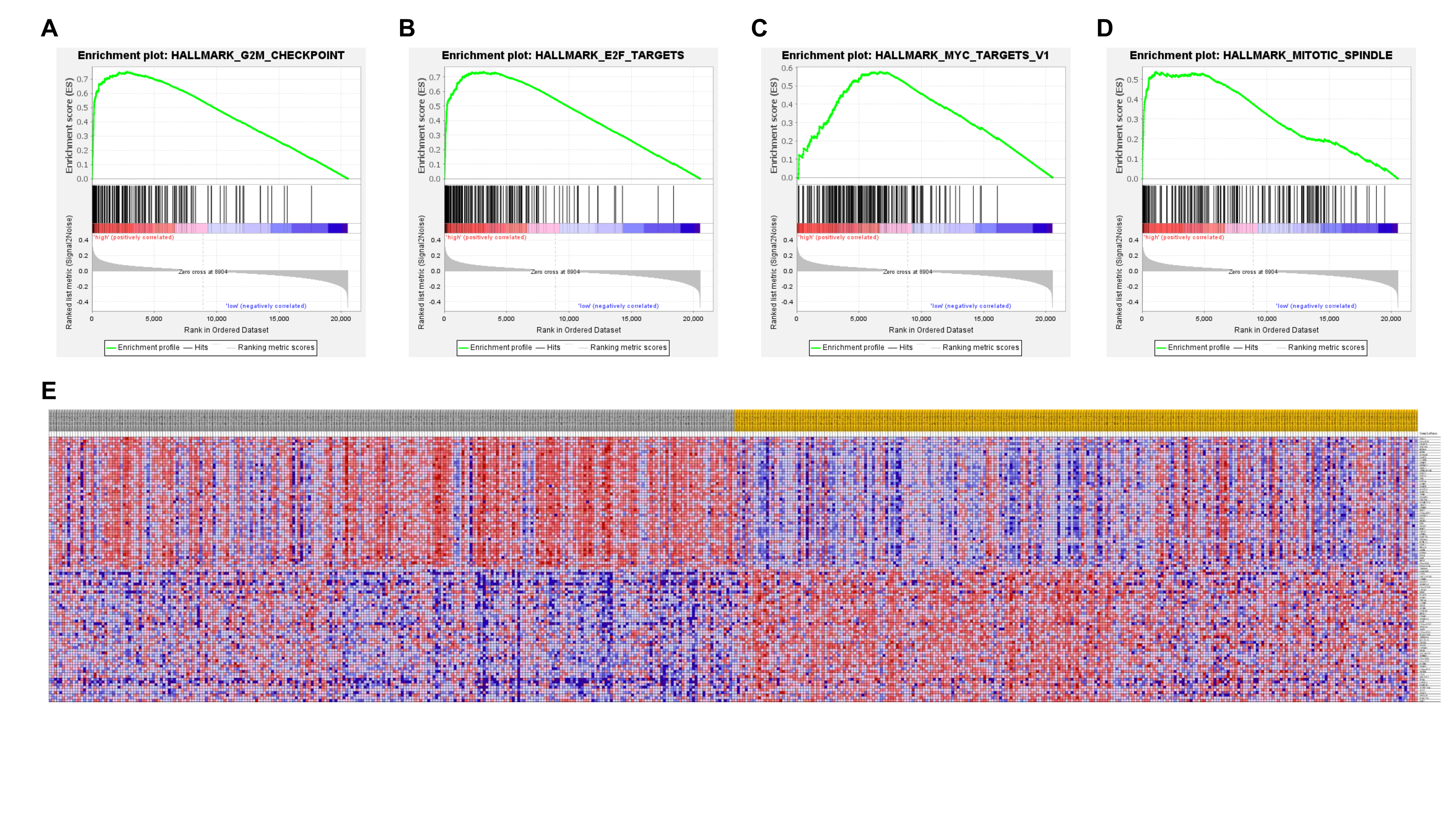

Supplement: Supplementary file 3 — Supporting information. [file CAI2-1-328-s004.tif]
