# Supplementary material for: Comprehensive analysis of the prognostic impact and immune implication of KIAA1429 in lung adenocarcinoma
Source: Cancer Innov. 2022 Dec 16;1(4):328–43. doi: 10.1002/cai2.40 (PMC10686173; doi:10.1002/cai2.40)
Supplement: Supplementary file 4 — Supporting information. [file CAI2-1-328-s001.docx]

**Table S1. Clinical characteristics of the LUAD patients with neoadjuvant immunotherapy (NCC cohort 2)**

| Characteristics | Patients (n = 13) |
| --- | --- |
| Age, years |  |
| <60 | 6 |
| >60 | 7 |
| Sex |  |
| Male | 8 |
| Female | 5 |
| Smoking history |  |
| Yes | 7 |
| No | 6 |
| Stage |  |
| II | 5 |
| III | 8 |
| Response evaluation |  |
| CR | 0 |
| PR | 4 |
| SD | 9 |
