# Supplementary material for: Comprehensive analysis of the prognostic impact and immune implication of KIAA1429 in lung adenocarcinoma
Source: Cancer Innov. 2022 Dec 16;1(4):328–43. doi: 10.1002/cai2.40 (PMC10686173; doi:10.1002/cai2.40)
Supplement: Supplementary file 6 — Supporting information. [file CAI2-1-328-s003.docx]

**Table S1** Correlation analysis between KIAA1429 and relate genes and markers of immune cells in TIMER

| Description | Gene markers | LUAD | | | | |
| --- | --- | --- | --- | --- | --- | --- |
|  |  | None | |  | Purity | |
|  |  | Cor | P |  | Cor | P |
| CD8+ T cell | CD8A | 0.125 | *** |  | 0.163 | *** |
|  | CD8B | 0.064 | *** |  | 0.081 | *** |
| T cell (general) | CD3D | -0.027 | *** |  | -0.002 | *** |
|  | CD3E | 0.034 | *** |  | -0.075 | *** |
|  | CD2 | 0.024 | *** |  | 0.057 | *** |
| B cell | CD19 | -0.054 | *** |  | -0.027 | *** |
|  | CD79A | -0.013 | *** |  | 0.023 | *** |
| Monocyte | CD86 | 0.120 | *** |  | 0.154 | *** |
|  | CSF1R | 0.123 | *** |  | 0.150 | *** |
| TAM | CCL2 | 0.051 | *** |  | 0.076 | *** |
|  | CD68 | 0.079 | *** |  | 0.104 | *** |
|  | IL10 | 0.073 | *** |  | 0.101 | *** |
| M1 Macrophage | NOS2 | 0.124 | *** |  | 0.130 | *** |
|  | IRF5 | 0.057 | *** |  | 0.066 | *** |
|  | PTGS2 | 0.114 | *** |  | 0.120 | *** |
| M2 Macrophage | CD163 | 0.255 | *** |  | 0.293 | *** |
|  | VSIG4 | 0.067 | *** |  | 0.083 | *** |
|  | MS4A4A | 0.076 | *** |  | 0.103 | *** |
| Neutrophils | CEACAM8 | -0.04 | *** |  | -0.04 | *** |
|  | ITGAM | 0.078 | *** |  | 0.106 | *** |
|  | CCR7 | -0.004 | *** |  | 0.027 | *** |
|  | KIR2DL1 | -0.002 | *** |  | -0.002 | *** |
|  | KIR2DL3 | 0.095 | *** |  | 0.117 | *** |
|  | KIR2DL4 | 0.164 | *** |  | 0.177 | *** |
|  | KIR3DL1 | 0.031 | *** |  | 0.027 | *** |
|  | KIR3DL2 | 0.111 | *** |  | 0.131 | *** |
|  | KIR3DL3 | 0.049 | *** |  | 0.051 | *** |
|  | KIR2DS4 | 0.056 | *** |  | 0.061 | *** |
| Dendritic cell | HLA-DPB1 | 0.140 | *** |  | -0.139 | *** |
|  | HLA-DQB1 | -0.146 | *** |  | -0.142 | *** |
|  | HLA-DRA | -0.102 | *** |  | -0.098 | *** |
|  | HLA-DPA1 | -0.055 | *** |  | -0.045 | *** |
|  | CD1C | -0.188 | *** |  | -0.185 | *** |
|  | NRP1 | 0.322 | *** |  | **0.324** | *** |
|  | ITGAX | 0.034 | *** |  | 0.059 | *** |
| Th1 | TBX21 | 0.091 | *** |  | 0.131 | *** |
|  | STAT4 | -0.006 | *** |  | 0.015 | *** |
|  | STAT1 | 0.387 | *** |  | **0.429** | *** |
|  | IFN-γ (IFNG) | 0.105 | *** |  | 0.134 | *** |
|  | TNF-α (TNF) | 0.078 | *** |  | 0.108 | *** |
| Th2 | GATA3 | 0.112 | *** |  | 0.155 | *** |
|  | STAT6 | 0.105 | *** |  | 0.106 | *** |
|  | STAT5A | 0.128 | *** |  | 0.158 | *** |
|  | IL13 | 0.073 | *** |  | -0.057 | *** |
| Tfh | BCL6 | 0.113 | *** |  | 0.109 | *** |
|  | IL21 | 0.207 | *** |  | 0.219 | *** |
| Th17 | STAT3 | 0.378 | *** |  | **0.375** | *** |
|  | IL17A | 0.002 | *** |  | 0.009 | *** |
| Treg | FOXP3 | 0.098 | *** |  | 0.143 | *** |
|  | CCR8 | 0.237 | *** |  | 0.289 | *** |
|  | STAT5B | 0.364 | *** |  | **0.367** | *** |
|  | TGFβ (TGFB1) | 0.116 | *** |  | 0.136 | *** |
| T cell exhaustion | PD-1 (PDCD1) | 0.093 | *** |  | 0.127 | *** |
|  | CTLA4 | 0.076 | *** |  | 0.120 | *** |
|  | LAG3 | 0.066 | *** |  | 0.093 | *** |
|  | TIM-3 (HAVCR2) | 0.071 | *** |  | 0.102 | *** |
|  | GZMB | 0.129 | *** |  | 0.164 | *** |
